# The Dark Side: Photosensitizer Prodrugs

**DOI:** 10.3390/ph12040148

**Published:** 2019-10-04

**Authors:** Sara Sansaloni-Pastor, Jordan Bouilloux, Norbert Lange

**Affiliations:** School of Pharmaceutical Sciences, University of Geneve, 1206 Geneva, Switzerland; Sara.SansaloniPastor@unige.ch (S.S.-P.); Jordan.bouilloux@unige.ch (J.B.)

**Keywords:** quenching, prodrugs, photodynamic therapy, 5-aminolevulinic acid

## Abstract

Photodynamic therapy (PDT) and photodiagnosis (PD) are essential approaches in the field of biophotonics. Ideally, both modalities require the selective sensitization of the targeted disease in order to avoid undesired phenomena such as the destruction of healthy tissue, skin photosensitization, or mistaken diagnosis. To a large extent, the occurrence of these incidents can be attributed to “background” accumulation in non-target tissue. Therefore, an ideal photoactive compound should be optically silent in the absence of disease, but bright in its presence. Such requirements can be fulfilled using innovative prodrug strategies targeting disease-associated alterations. Here we will summarize the elaboration, characterization, and evaluation of approaches using polymeric photosensitizer prodrugs, nanoparticles, micelles, and porphysomes. Finally, we will discuss the use of 5-aminolevulinc acid and its derivatives that are selectively transformed in neoplastic cells into photoactive protoporphyrin IX.

## 1. Introduction

Nowadays, personalized medicine is receiving attention from many research groups worldwide. Different approaches have been developed, leading to more specific and efficient treatments. One method in this context is photodynamic therapy (PDT). This therapeutic approach has high versatility and chemical possibilities, low adverse effects, repeatability, and the possibility to be combined with other treatments. These properties, among other advantages, provide interesting properties for cancer treatments.

**Figure 1:**
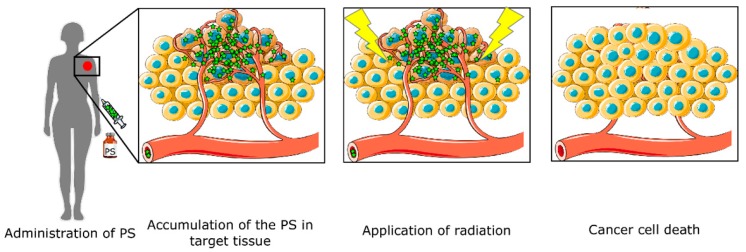
Photodynamic therapy (PDT) diagram. The therapy starts with photosensitizer (PS) administration. The drug is accumulated in the target tissue, and only when irradiated with light does leads to cell death.

PDT is based on three different non-toxic factors: a light-activated drug (photosensitizer, PS), local irradiation, and molecular oxygen. The process is carried out following these steps (Figure 1): PS application, internalization and accumulation in the target tissue, and irradiation.

Only cells that have internalized the PS are located in the irradiation area and contain enough molecular oxygen will be affected by the cytotoxic reactive oxygen species (ROS).

One of the main advantages of PDT is the possibility to combine this treatment with other complementary techniques [1]. For instance, combinations with photothermal therapy (PTT), chemotherapy, or immunotherapy have been reported [2,3,4,5,6,7,8].

**Figure 2:**
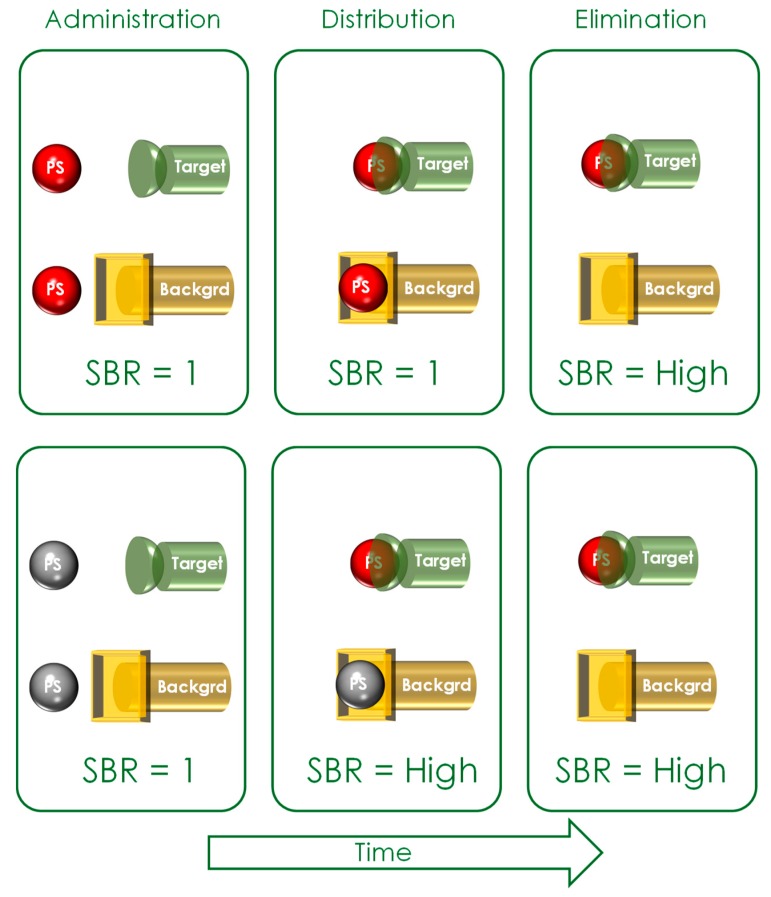
Improving the signal-to-background ratio (SBR): Upper row: Intrinsically active targeted photosensitizers (PSs) have a low SBR during administration and distribution within the body. Only during the elimination phase does the SBR become higher. Lower row: Optically silent PSs already have a high SBR during the distribution phase. Red spheres represent photoactive photosensitizers; Grey spheres represent non-active photosensitizers.

Historically, the first PDT compounds employed were hematoporphyrin (HP) and hematoporphyrin derivatives (HpD) [9,10,11,12]. Second-generation PSs were aimed at increasing the therapy’s selectivity and improving their pharmacokinetic and chemical properties. Furthermore, targeting strategies such as encapsulation into nanoparticles or micelles, or chemical coupling to antibodies were tested in order to increase the selectivity to target tumor cells [13,14]. However, the main current issue with the suggested targeting strategies is the intrinsic PS photoactivity. Due to this limitation, the most promising strategy to improve PS selectivity is to develop compounds that are optically silent until reaching their respective target. Only when they reach and interact with the target do they become activated, thereby improving the signal-to-background ratio (SBR) (see Figure 2).

The PS silencing can be achieved with quenched photosensitizers or using pro-drugs with small molecular weights, such as 5-aminolevulinic acid (5-ALA) or its derivatives. These optically silent PS prodrugs are photodynamically inactive until they encounter the proper chemical or biological trigger, inducing a conformation change and becoming photodynamically active. Such chemical or physiological triggers include (1) pH, (2) redox potential, (3) enzymatic activities, (4) DNA, or (5) temperature. This review is dedicated to the development and testing of such photosensitizer prodrugs.

## 2. Quenched Photosensitizers

Quenched PSs are designed to limit the cytotoxic oxygen species production to the target tissue, controlling the generation rate and the location. The principle behind the quenching effect relies on the rapid transfer of energy between PSs and another dye molecule or optical nanomaterial. This transfer occurs through two different mechanisms: long distance interaction (dynamic quenching) (10–100 Å) such as fluorescence resonance energy transfer (FRET) quenching, or short distance (1–10 Å) exchange such as static quenching or exciplex formation [15].

The regulation of the photo-induced electron transfer effect (PET), the resonance energy transfer (RET), the self-quenching, and intersystem crossing also represent a potential approach for the design of switchable PSs [16]. The PET ROS regulation principle requires another simultaneous process such as intersystem crossing. A donor and an acceptor of electrons will form this system [17]. In addition, it is important to know that RET, FRET, and exciting energy transfer (EET) are methods that depend on the distance between the acceptor and donor. Consequently, in those strategies the donor and the acceptor are usually linked [17].

The deactivation of the PS’s excited singlet-state, thanks to the quencher interaction, reduces the number of excited PS molecules available to enter into the triplet state and consequently lower the singlet oxygen generation [18]. Furthermore, quenched constructs seem to be resistant to photobleaching, resulting in an increase in the PS fluorescence after extended tissue irradiation [18].

Multiple approaches have been developed in order to silence PSs. Some researchers designed sensitive PSs for which the activity is dependent on different cellular factors such as pH or ion concentration. Other groups have been working on developing PSs covalently attached to carbon nanotubes, carotenoids, or commercially available black hole quenchers (BHQs). Finally, self-quenching of the photosensitizer is another route for activity control [19].

In many self-quenched prodrugs approaches, multiple PSs are linked to a polymeric carrier such as polylysine, glycol chitosan, hyaluronic acid, dextran, pullulan, or heparin. Also, some nanoplatforms have been shown to be efficient carriers, such as graphene oxide, single walled carbon nanotubes, and gold nanorods [20]. The drug activation requires PS separation from the carrier, triggered by a specific stimulus from the target cell.

Another useful carrier is hydrophilic poly(oligoethylene glycol) methyl ether methacrylate (POEGMA), which is a thermo-sensitive polymer. This structure has different hydrogen binding sites and has an increased chain flexibility [21]. POEGMA has been widely used as a delivery system because it is economical, non-toxic, non-immunogenic, water-soluble, and biocompatible [22].

Undesired quenching effects also have been reported, such as PS aggregation-induced self-quenching, related to proximity and interactions with reference to fluorescence and singlet oxygen (^1^O_2_) generation reduction [23]. Bae et al. took advantage of this principle using a self-quenched polysaccharide-based nanogel, finding reduced photoactivity in circulation due to PS aggregation [24].

Another quenching approach employs multiple PSs covalently linked to a unique polylysine chain. Some studies have reported that the complex fluorescence emission is directly related to the PSs number attached to the matrix [25,26]. These compounds showed up to 600 times less fluorescent quantum yields compared to the active compound. However, the major problem is their reduced solubility with high PS loading. For instance, Campo et al. developed a complex of 30 PSs per chain, but they were unable to test it due to their low solubility. Aiming to solve this drawback, some strategies have been designed such as linking a hydrophilic chain to the PSs instead of the currently employed hydrophobic PSs, modifying the polymeric backbone, and/or decreasing the overall molecular weight [26]. The main drawback of these first-generation polymeric PS prodrugs is the non-specific reactivation mediated by proteases that recognize lysine–lysine motifs.

Recent studies have been focused on developing a new strategy to improve the solubility of these large complexes, linking PSs prodrugs through a specific peptide linker which is protease sensitive [27]. Examples are the polymeric PS prodrugs designed for the overexpression of thrombin or urokinase-like plasminogen activator [28,29]. While the thrombin-sensitive polymeric prodrug showed significant activities in arthritic mice, the latter was used to selectively treat xenografted mice with cells from prostate cancer origin, showing specific activities *in vitro* and *in vivo*. Nevertheless, these polymeric PS prodrugs have large polydispersity, reproducibility, and an unknown position and number of PS moieties per polymeric chain.

**Figure 3:**
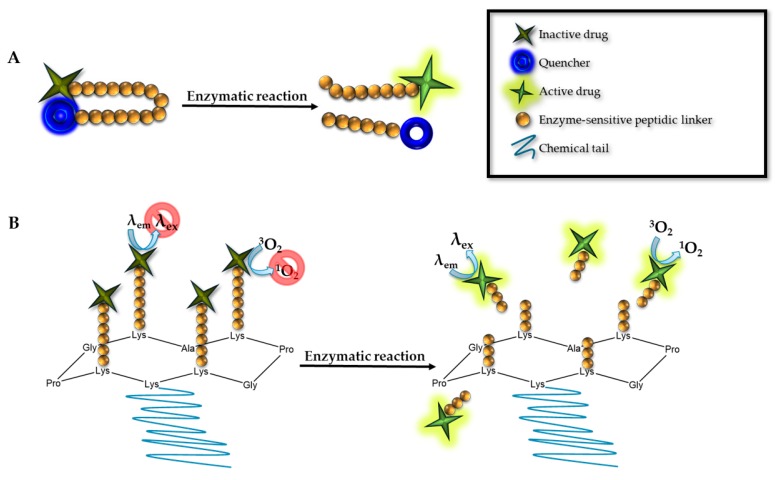
(**A**) Molecular beacon. In its initial conformation, the PS is quenched thanks to the proximity between the PS and quencher linked to a synthetic chain, which could be a peptide, DNA, or RNA. After the proper digestion or complement sequence hybridization, the PS is activated by the physical separation between the two components. (**B**) Regioselectively-Addressable Functionalized Templates (RAFTs). The molecular structure is based in a cyclopeptidic scaffold. We can differentiate two main domains, one functionalized with different PSs, and the second domain containing a chemical chain to increase the solubility. In its initial conformation, the molecule is quenched, until the linker encounters a specific digestion, becoming active.

In order to overcome the polymeric PS disadvantages, our research group recently tested Regioselectively-Addressable Functionalized Templates (RAFTs) as carriers for quenched PSs (Figure 3). RAFTs are cyclopeptidic scaffolds designed to provide a tailored attachment and spatial separation of different biologically active moieties, allowing functional moieties attached to the upper and lower part of the RAFT scaffold. Orthogonal chemistry allowed us to selectively tether up to three different chemical moieties to the RAFTs. The best results with respect to the quenching capacity were achieved with the combination of BHQs with two PSs. The BHQ containing cyclopeptidic PSs prodrugs was 700 times less fluorescent than the unquenched PSs. Furthermore, we were unable to detect singlet oxygen production. The linkers can be sensitive to some target tissue stimulus, reducing the off-target effects of the PDT [30]. We have developed linkers sensitive to proteolytic activities that are often overexpressed in tumor tissues [28,31,32].

The molecular beacon (Figure 3) is a PS linked to a quencher; the linker is degradable or can change the configuration. They offer a control over the fluorescence emission. In the molecular beacon approach, a quencher/donor pair is positioned in each extreme of a synthetic oligonucleotide chain, DNA, RNA or a peptide. In absence of the complementary DNA or RNA sequence or the specific proteases, the PS will be silenced. Only in the presence of the proper nucleotide chain or protease, the beacon complex will change its conformation, leading to the PS activation. Physically separating the donor from the acceptor restores the PS photoactivity. [33,34,35,36].

There are different enzymes to degrade their respective linkers, for instance exopeptidase. Chiba et al. designed an aminopeptidase-degradable linker, activating the related PS. This complex was then activated by ɣ-glutamyltranspeptidase 2, a peptidase overexpressed in different kinds of tumors such as ovarian, lung, and prostate cancers [37].

Besides proteolytic activities, other physiological triggers can be used to activate quenched PSs. Tumors show lower pH values than normal tissues [38]. Therefore, PS prodrugs can be designed to respond to a pH gradient, promoting a change in the protonation and aggregation tendency of the compounds [39]. For instance, LDH-ZnPcS8 has excellent tumor pH-responsive properties, including a high quenching effect in normal tissues and at the same time a low *in vivo* skin phototoxicity [40].

Redox balance is essential for all cell kinds. However, tumors have an altered balance compared to healthy cells. This differential opens the door to design new sensitive PS prodrugs, for instance using desulphated linkages like the ones used commonly for drug delivery [2,16].

As mentioned before, oligonucleotides can be linkers. When the complementary sequence is present and interacts with the complex, the molecular beacon will be disassembled. Li et al. worked in an oval-shaped nanoassembly responding to nucleic acids. The main structure is composed of a PS and a drug (mitoxantrone) attached to the carrier’s structure [41].

The following table (Table 1) shows different examples of PS prodrugs for different types of stimuli.

## 3. Nanoparticles

Nanoparticles are sub-microscopic particles with sizes between 1 and 500 nm. The interest in these particles is based on their versatility and the possibilities of improving some PS characteristics such as their selectivity, bioavailability, pharmacokinetics, and cell uptake. Furthermore, they enable control of PS aggregation, protection of the PS from deactivation, specificity for the target, decreased toxicity, and provide a multifunctional delivery platform.

As mentioned before, there are differential parameters between healthy and non-healthy tissue, which can control drug activation. Nanoparticle-mediated delivery can be based on passive or active targeting strategies. The first depends on the tumor tissues permeability and retention. Tumors show poor lymphatic drainage, making feasible a larger nanoparticle proportion in non-healthy tissues by the passive approach [48]. The active targeting is dependent on nanoparticle stimuli activation.

Some nanoparticles have been designed with biodegradable polymers, which respond to environmental changes, leading to degradation and drug release. The main degradation stimuli are pH, metabolite concentrations such as glucose and different element concentrations as oxygen, ions, redox potential, enzymes, etc. [49]. Wei et al. synthetized a pH-sensitive nanoparticle in which activation depends on the decomposition of the metal–phenolic network in an acidic environment. Additionally, they attached folic acid to the nanoparticles to facilitate the targeting of tumor cells [50].

Nanoparticles active targeting can also be achieved with short DNA or RNA chains that bind different targets like proteins (transmembrane, intracellular, and soluble), carbohydrates, or small drugs. Another alternative is the use of aptamers, short nucleotide chains with well-defined three-dimensional architectures. Aptamers can bind proteinic and non-proteinic targets. Furthermore, their small size provides a lack of immunogenicity. When the aptamer binds the specific target, a structural change is induced, allowing the drug release. The main problem of this approach is the aptamer susceptibility to the nuclease degradation. Nevertheless, some studies proposed nucleotide chemical modification to avoid their fast degradation [51,52].

One successful aptamer example was studied in breast cancer by Yen-An Shieh et al., who showed that the affinity of these nanoparticles to breast cancer cells was increased compared to normal cells [53].

Another example is the targeting of some nutrient receptors overexpressed in cancer cells have compared to normal cells [54].

Brevet et al. developed mesoporous silica nanoparticles linked to mannose moieties, targeting receptors overexpressed in breast and prostate cancers. With these compounds, a higher PDT efficiency *in vitro* and *in vivo* was observed. Interestingly, the PSs encapsulated into the mannose nanoparticle were quenched and only activated by the proper stimulus [55,56,57,58].

Folic acid-laden nanoparticles have been also studied targeting the folate receptor, which is upregulated in some cancers but absent in most healthy tissues. The study reported by Idris et al. showed an improvement of PDT efficacy *in vitro* and *in vivo* employing this target [59]. In addition, other studies such as that by Huang et al. demonstrated acid folic efficiency as a target in stomach cancer, showing an increase of PS accumulation in tumors cells, with low cytotoxicity and good solubility of the nanoparticles [60].

### 3.1. Nanoparticles Used for Silenced PS

#### 3.1.1. Metal Nanoparticles

Metal nanoparticles, mainly composed of gold, silver, and platinum, have been extensively studied. Their common characteristic is an important quenching effect, produced by the localized surface plasmon resonance effect. When PSs are loaded inside the metal surface, their electrons interact with the nanoparticle plasmon field. Due to this interaction, PSs are initially quenched during the drug delivery, and only after being released into the target tissue the drug will become active.

Gold nanoparticles have been widely used for PS delivery due to their appropriate biological compatibility, multiple functions, easy chemical modification, simple preparation, and their inherent strong surface plasmon resonance absorption band located in the near infrared region. Furthermore, these nanoparticles can be used in combination with photothermal therapy (PTT) [61,62]. Additionally, they can be designed to bind specific ligands such as proteins, DNA, peptides, or sugars [63]. However, they are not biodegradable and their elimination mechanism are still unknown [61].

An interesting study led by the laboratory of El-Hussein compared the efficacy of gold and silver nanoparticles in A549 and MCF-7 cell lines. In this article they observed how silver nanoparticles elicit a greater photodynamic effect than gold nanoparticles alone [64].

Other groups such as Qiu et al. developed gold nanoparticles conjugated to quenched PSs. In addition, they improved the nanoparticle cell-internalization, conjugating them with activatable cell-penetrating peptides. This structure was studied *in vitro* and *in vivo*, showing a reduced systemic toxicity and tumor growth inhibition [62].

#### 3.1.2. Polymeric Nanoparticles

The polymeric particles include organic and inorganic nanoparticles, which can have biodegradable properties. Recent studies highlight their biocompatibility, simple preparation and interesting bio-mimetic character [49].

The polymeric vehicles can be presented as a polymeric micelle, nanosphere or nanocapsule. The most employed polymers include poly(hydroxyalkanoates) (PHAs) or synthetic polymers like poly(orthoesters), poly(β-amino esters) (PbAE) and poly(α-hydroxy esters). The last group includes poly(D,L lactic acid) (PLA), poly(glycolide) (PGA) and poly(ε-caprolactone) (PCL) [61].

Encapsulation of PSs by entrapment, dispersion or adsorption showed and improved solubility, pharmacokinetic properties and polymeric particles payload [61]. These improvements allow a much more adjustable PS release compared to other delivery systems. Furthermore, this methodology permits a long-term release combined with a short period of burst, increasing the therapy possibilities

These polymeric nanoparticles can also be designed to quench the PS’s activity, reducing the ROS production in the non-specific target. There are different possibilities to do so, following the previously described strategies (Section 2).

For instance, Zeisser-Labouèbe et al. studied the possibilities of PLA and PLGA nanoparticles for hypericin delivery. Their PS does not show any dark toxicity in a free or encapsulated state when studied *in vitro*. They showed different efficiencies after testing each loaded nanoparticle, showing a significant higher efficiency with PLA vehicles compared with PLGA or free hypericin [65]. After further studies, they demonstrated that ROS production after light irradiation decreased by loading the PS inside their PLA nanoparticle. In addition, they observed that when the PS encapsulation rate was faster, a more efficient PS deactivation was promoted [66].

Vargas et al. designed nanoparticles formulated with poly(D,L-lactide-co-glycolide) and employed m-THPP as a PS. In order to test this new structure, they used the chorioallantonic membrane model, showing no phototoxicity. Furthermore, they studied different batches with different particles sizes. The smallest particle was reported to have the greatest *in vivo* activity, the highest ROS formation, and the fastest PS release *in vitro* [67]. Another interesting study employing poly(lactic-co-glycolic acid) vehicles was led by McCarthy et al. They reported good nanoparticle stability, PSs with quenched excited states after encapsulation (which is released and activated inside the cell), and nontoxic side-effects after systemic administration *in vivo*. In addition, they reported complete cancer eradication in mouse models [68].

The polymer (PPF-Ir-g-(POEGMA-b-PGal)) of Lu et al. after light irradiation showed an efficient xenograft Hep G2 tumor inhibition and a high apoptosis level *in vitro*. At the same time this polymer showed a low dark toxicity *in vivo* [69].

Dendrimer designs have been gathering great interest in relation to polymeric nanoparticles. This structure allows control of size (total size 1–10 nm), functional groups on their branches and number of modifiable areas that can be used to obtain new derivatives and lipophilicity. At the same time, these derivations can also facilitate intracellular accumulation and decreased PSs toxicity, as was observed in recent articles [61,70].

Dendrimers can also be assembled with copolymers to obtain micelles, prolonging the average blood circulation time, increasing the accumulation in tumors and the singlet oxygen generation, while decreasing aggregate formation [71].

Some examples of new dendrimers nanoparticles approaches include that of Oar et al. who developed dendrimers showing a >99% quenching effect on the donor emission in aqueous media. Another example is the work of Li et al. They studied the feasibility of using dendrimers to deliver indocyanine green and hematoporphyrin. The PS was quenched until the application of a 808 nm laser, becoming active and starting the PTT effect. The quenched effect of this experimental design was related to the indocyanine green component and its distance from the hematoporphyrin. With this design they demonstrated significant cell apoptosis with ROS generation after laser activation using an *in vitro* model [72].

#### 3.1.3. Carriers Based on Lipids

There are different components that can be employed to produce a lipid-based carriers, including liposomes, polymersomes, micelles, and porphysomes.

(1) Liposomes

Liposomes are built with mono or multiple concentric bilayer membranes structures and can be derived from natural or synthetic lipids. These liposomes are able to contain and transport drugs in their core (hydrophilic PSs) or in their lamellae (hydrophobic PSs) [73].

One of the problems of these vehicles is the cholesterol proportion. Cholesterol is employed to increase the membrane rigidity of the carrier, but at the same time it reduces the permeability of the encapsulated PS. The second problem with these nanoparticles is the easy exchange of lipids between the nanoparticle and some high-density lipoproteins, leading to nanoparticle disintegration and then to the early drug release. The described problems significantly reduce the circulation time of particles, also showing a reduced uptake compared to other delivery options [61].

Moreover, it is possible to conjugate the liposome with other molecules to achieve a more specific delivery, such as peptides or antibodies, among others. Furthermore, these conjugated liposomes can be also designed to be specifically activated by different mechanism from the target cell, such as change in pH, enzyme concentration, etc. An example of activatable liposome was described by Feng et al. using a near-red light stimulation. The quenched effect was induced by the fluorescence resonance energy transfer (FRET) and studied *in vitro* and *in vivo*. In addition, they studied skin photo-toxicity, and found no side effects after irradiation [74].

(2) Polymersomes

Another interesting lipid-based carrier is the polymersome. These are structurally similar to the liposomes but made of amphiphilic synthetic polymers. At the same time, one polymersome can be built of different blocks types, resulting in near endless possibilities of combinations. These lipid nanoparticles can also be functionalized to target specific tissues, reducing the off-target distribution. An example of a targeted polymersome is described in Hou et al.’s work. They tested a hydrophobic upconverting nanoparticle core with a hydrophobic polymersome shell and with or without targeting peptides *in vitro*. The peptide-labeled nanoparticles showed a lower cytotoxicity and increased the cellular uptake. At the same time they were efficient killing the cancer cells after light irradiation [75].

(3) Micelles

Micelles consist of self-assembled closed lipid monolayers with a hydrophobic core and hydrophilic shell [54]. In this area, Li et al. for example worked with micelles composed of a chitosan derivative, which were not cytotoxic before irradiation *in vitro*. Their results suggested that the tested micelles were efficiently delivered to cultured cells. In order to determine the drug internalization, they analyzed the evolution of the fluorescence on the cultured cells as their micelles showed a higher fluorescent level than the free PS, facilitating the analysis [76].

Micelles can also be designed as switchable carriers sensitive to an external stimulus, such as for instance singlet oxygen or light irradiation. Furthermore, the size of micelles can be easily modulated when replacing imidazole by hydrophilic urea, leading to a controlled PS release. On example of a complex micelle based approach was demonstrated by Koo et al. The created pH-responsive polymeric micelles had a significant PDT efficiency in 4T1 tumor-bearing mice and no dark toxicity *in vitro*. In addition, they also demonstrated the utility of this approach for tumor diagnosis [77].

(4) Porphysomes

Porphysomes are nanovesicles derived from porphyrin-lipid bilayers. They show a high static quenching effect induced by their composition. Porphysomes have a structure and loading capacity similar to liposomes. Interestingly they have a high porphyrin payload, advantageous for a good PDT and fluorescent imaging agent. They are also biocompatible, non-toxic and biodegradable. In addition, it is possible to attach different molecules to the porphysomes structure in order to increase their specificity for the target tissue.

Lovell et al. developed new porphysomes that showed a higher quenching capacity than the observed with PS located inside a simple bilayer, showing that the porphyrin–lipid orientation promotes porphyrin interaction and quenching. Moreover, after loading a low porphyrin density into the carrier, they observed that no significant fluorescence quenching was induced, explained by a self-quenching effect. Furthermore, they also demonstrated *in vivo* the self-quenching effect using mice bearing KB cell xenograft. However, after 2 days there was a high tumor fluorescence signal that should be studied further [78].

Another example of a successful porphysome design was reported by Ng et al. They tested a self-sensing porphysome, known as the FRETysome, employing bacteriopheophorbide as a fluorescent energy acceptor. Due to this design, the carrier was successfully imaged after the subcutaneous injection in nude mice. In addition, they also observed the persistence of nanovesicles at the tumor site after 24 h and 48 h post-injection [79]. Another example of the diagnostic utility of these nanoparticles was shown by Philp et al., who used porphysomes to guide the detection and real-time resection of primary tumors, lymph nodes and abdominal metastasis in rabbits [80].

Xu et al. used a glutathione-responsive supramolecular porphysome to guide the drug release (DOX) by fluorescence. Their cytotoxic assay demonstrated that cancer cells efficiently internalize the derived nanovesicles [81]. Finally, porphysomes can also encapsulate MRI contrast agents. Huynh et al. encapsulated a fluorinated gas in photonic microbubbles produced from porphyrin-lipid components with a porphyrin shell [82].

## 4. 5-ALA and Derivatives

5-Aminolevulinic acid (5-ALA) is a natural metabolite present in every cell and is the main precursor of porphyrines. 5-ALA is a zwitterionic molecule at physiological pH, exhibiting hydrophilic properties that drastically reducing its ability to move through biological tissues barriers [83]. It has been shown that following exogenous administration of 5-ALA, high concentrations of the PS protoporphyrin IX (PPIX) in neoplastic tissue can be observed.

Treatments with 5-ALA usually follows the oral, parenteral, or topical administration. After the oral application, 5-ALA’s bioavailability is lower compared with to the intravenous administration, and for that reason a higher oral doses are is required to achieve the same final PPIX concentration [84]. However, high 5-ALA doses are related to some side effects such as nausea, vomiting, liver function problems, and decreased systolic and diastolic blood pulmonary pressure [85,86].

### 4.1. Synthesis of 5-ALA

The 5-ALA biological synthesis is divided in two possible pathways. The first pathway is known as the C4 or Shemin pathway, located inside the mitochondria. This pathway ends with the glycine and succinyl-CoA condensation, catalyzed by the *ALA synthase* (ALAS). The second pathway, known as C5, is present in plants, algae, and several bacterial species. It consists of three reactions: the conversion of glutamate to glutamyl-tRNA by *Glutamyl-tRNA synthetase,* the transformation to glutamate-1-semialdehyde by *glutamyl-tRNA reductase HemA,* and the conversion into 5-ALA by *glutamate-1-semialdehyde amino transferase HemL* [87,88,89].

**Figure 4:**
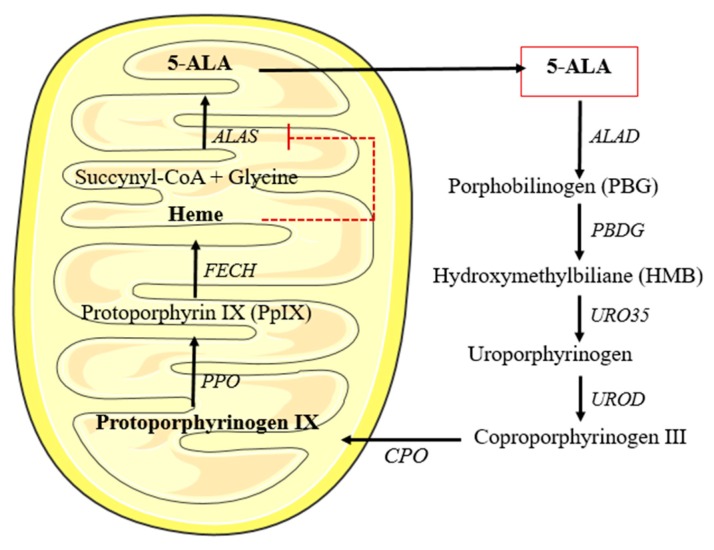
Heme biosynthesis. 5-aminolevulinic acid (5-ALA) resulting from the condensation of succynil-CoA and glycine. It can pass through the mitochondrial membrane. Once inside the cytosol after different enzymatic reactions it is converted into coproporphyrinogen III, which in turn re-enters the inner mitochondrial membrane. Then, after two consecutive reactions it is transformed into heme. This heme group works functions as a negative feedback for the 5-ALA synthesis.

In mammalian cells, 5-ALA is principally be transformed into heme, from which the production of 5-ALA (by *ALAS*) is feedback-regulated (see Figure 4). However, when 5-ALA is exogenously administered there is enzyme saturation in the route and the cell is unable to convert all the PPIX to heme, causing an overproduction and accumulation of this route intermediary, which is at the same time a natural PS (PPIX) [90].

### 4.2. Specific PPIX Accumulation in Cancer Cells

The explained PPIX accumulation after 5-ALA supply occurs in healthy and cancer cells; nevertheless, some factors have been suggested to induce the differential PPIX accumulation observed between cancer and healthy cells [90]: (1) Availability of iron, (2) lower activity of *ferrochelatase,* (3) transferrin receptor expression, (4) altered expression of enzymes in heme biosynthesis, and (5) mitochondrial content. The principal hypothesis for the selective accumulation of PPIX after administration of 5-ALA have been discussed in detail recently [91]. Furthermore, it has been mentioned that modern tools in microbiology have not been used to determine the reasons for this phenomenon. However, in the context of this review, it has to be mentioned that 5-ALA induced PPIX is an ideal silenced PS, since none of the intermediates or products in heme biosynthesis is photoactive.

### 4.3. 5-ALA Derivatives

After the discovery of the selective accumulation of PPIX following administration of 5-ALA in neoplastic cells, a real boost in this research area appeared in the late 1980s. However, early on it seemed clear that only sub-optimal doses of PPIX can be achieved using acceptable doses of 5-ALA. The main drawbacks related to 5-ALA-mediated therapy are the limited uptake, low solubility, and the fast pro-drug elimination, resulting in a low substrate availability in the cytosol. Thus, strategies such as encapsulation into liposomes [92,93,94], addition of iron chelating agents [92,95,96,97,98], and addition of cell penetrating agents such as DMSO [99,100] have been tried without clinically relevant success.

In order to increase the uptake of this substrate for heme biosynthesis about 20 years ago, the first 5-ALA derivatives have been reported [83,101,102,103]. In the beginning, research focused on the simple more lipophilic carboxylic ester of 5-ALA. These ester derivatives could be linear, branched, cyclic, etc. Ester derivatives are only available for topical (cream, gel, ointments, or solutions) and other non-systemic applications, because due to their toxicity and low bioavailability they are not convenient for systemic application [104,105]. An interesting approach of 5-ALA derivatives has recently been published [106]. A 5-ALA derivative carrying butyric acid in order to increase heme formation in anemic patients was reported.

Another way to modify 5-ALA’s structure is to create pseudo peptides by addressing the amino-function. The peptide derivation opens the door to the active transport through the membrane using dipeptide transporters. In addition, they are also relevant to target some tumor enzymes (aminopeptidases), which remove the peptide only when the derivative is internalized by the target cells, activating 5-ALA. Moreover, peptide derivatives are more stable at physiologic pH than their precursor.

One good example of peptide-derivatives is described in Giuntini et al. who synthetized different 5-ALA peptides derivatives, with a lipophilicity improvement and a more efficient cellular accumulation compared to 5-ALA [107]. However, since the amino-function is essential for the formation of the pyrrol ring, most of these pseudo 5-ALA peptides are less efficient in PPIX formation then 5-ALA esters.

However, recently we have developed phosphatase-sensitive 5-ALA esters with significantly lower acute systemic toxicity, than the corresponding 5-ALA esters [108]. However, *in vitro* they were as efficient in PPIX formation as their native ester. These compounds might play an essential role in the treatment or diagnosis of diseases where topical administration is not possible or difficult.

Another example of research focused on improving this characteristic is the work of Battah et al. They worked with dendrimers with up to 18 5-ALA residues attached. Those dendrimers demonstrated a more efficient PPIX synthesis than 5-ALA free, with minimal dark toxicity *in vitro* [109]. Recently we have reviewed the essential work on 5-ALA derivatives and their clinical implementation in detail [110]. Commercially available drugs containing 5-ALA and its derivatives are listed in Table 2.

In the last years, 5-ALA and its derivatives have been studied and employed to treat multiple diseases, showing their therapeutic potential.

These 5-ALA compounds have been tested to treat different kind of cancers such as idiopathic elastosis [111], ovarian clear-cell carcinoma [112], brain cancer [113], bladder cancer [114,115], lung cancer [116], Paget’s disease [117,118], and Bowen’s disease [119] among others.

5-ALA derived drugs and PDT have shown also to be effective to treat some virus-induced lesions. Some examples are recalcitrant foot and hands warts and epidermodysplasia verruciformis [120]. Additionally, PDT using 5-ALA has shown high effectivity in the treatment of fungus and bacteria resistant to antibiotics [121].

PDT is also a relevant tool for diagnosis *in vivo*, for instance to detect brain tumors [122], gliomas [123], endometriosis, intraepithelial lesions of the cervix, and lung cancer among others [124]

Finally, the last described application of the 5-ALA compounds, far from the field of medicine, is related to farming for use as a fertilizer, herbicide, and insecticide [125,126], and also facilitating plant tolerance to salt and cold temperatures [88].

## 5. Conclusions and Perspectives

In times where modern molecular biology constantly elucidates new mechanisms of cancer occurrence and progression, diseases will be detected at earlier stages in the future. Therefore, minimally invasive methods are needed to fight tumors at localized stages. In this context, PDT and PD might have an important role in treating or assisting surgery in the future. However, current photosensitizers lack selectivity for the diseased tissues and may potentially harm neighboring healthy tissues. Photosensitizer prodrugs provide the possibility to obtain high tumor-to-normal tissue ratios at early stages of the distribution phase of the pharmacokinetic profile of the prodrug. At the moment, only 5-ALA induced PpIX has obtained marketing authorization for the treatment or detection of different kinds of cancer. However, also other kinds of enzymatic alterations in cancer cell metabolisms could potentially be targeted in the future. This review aimed to illustrate different potential routes that can be followed. Furthermore, with expected advances in tumor biology, other mechanisms can be targeted to design different photosensitizer prodrugs.

## Figures and Tables

**Table 1:** PSs responsive to different stimuli. POEGMA: poly(oligoethylene glycol) methyl ether methacrylate.

Stimulus	PS	Phototoxicity	Imaging	Reference
**pH**	phthalocyanine	No	Yes	[42]
	BODIPY	-	-	[43]
**ɣ-Glutamyltranspeptidase**	hydroxymethyl selenorhodamine	Yes	Yes	[37]
**Temperature**	POEGMA	No	No	[44]
**Glutathione**	BODIPY	No	No	[19]
	BODIPY	Yes (High dark/photocytotoxicity ratio)	Yes	[45]
	Dendritic phthalocyanines	No	No	[46]
	BODIPY	No	No	[47]
**pH and thiol-responsive**	BODIPY	No	No	[20]

**Table 2:** Commercialized 5-ALA and derivatives.

Product Name	Photosensitizer	Administration	Treatment of	Skin Photosensitivity
**Levulan ^®^** **Kerastick ^®^**	5-ALA	Topical, oral, intravenous/powder for solution, cream	Actinic keratoses	1–2 days
**Effala ^®^/Alacare ^®^**	5-ALA	Medicated plaster	Actinic keratosis	
**Gliolan ^®^**	5-ALA	Powder for oral solution	Detection malignant glioma	
**Metvix ^®^**	5-ALA methyl ester	Cream, topical	Actinic keratosis Basal cell carcinoma Bowen’s disease	uncommon
**Hexvic ^®^**	5-ALA hexyl ester	Topical/powder for solution, gel	Detection of recurrent bladder cancer	uncommon
**Cysview ^®^**	5-ALA hexyl ester	Powder for solution	Detection of recurrent bladder cancer	
**Ameluz ^®^**	5-ALA	Cream, topical	Actinic keratosis	uncommon
